# A Manual of Medical Jurisprudence

**Published:** 1898-01

**Authors:** 


### Book Notices.

A MANUAL OF MEDICAL JURISPRUDENCE, by Alfred Swaine Taylor, M. D., F. R. S. Revised and edited by Thomas Stevenson, M. D., London, Fellow of the Royal College of Physicians of London; Lecturer on Medical Jurisprudence and on Chemistry at Guy's Hospital; Examiner in Forensic Medicine in the University of London; External Examiner in Forensic Medicine in the Victoria University; Official Analyst to the Home Office. Twelfth American; edited with citations and additions from the twelfth English edition, by Clark Bell, Esq., LL.D., President of the American International Medico-Legal Congress of 1889; President of the American International Medico-Legal Congress of 1893 (Chicago); President of the Medico-Legal Congress of 1895 (New York); Honorary Member of the Medico-Legal Society of France; of the Society of Mental Medicine of Belgium; Associate Member of the Society Medico-Psychologique of Paris; Corresponding Member of the Society of Psychiatry of St. Petersburg; of the Netherlands; of Lisbon, Portugal; of the Society of Freniatria of Italy; of the Belgian Society of Anthropology; and Ex-President of the Medico-Legal Society of New York. New edition. Price, cloth, $4.50; leather, $5.50. Lea Brothers & Co., New York and Philadelphia.

For many years Taylor's Medical Jurisprudence has been a favorite text-book, a standard authority, and the professions, both of law and medicine, will welcome the advent of a new, revised, and thoroughly up to date edition, especially as it has been revised and issued by Hon. Clark Bell, than whom there is not an abler medical jurist on either continent. Judge Bell has carefully scanned the last edition line by line—cutting out here and adding there, and in some instances rewriting entire chapters—until the volume now before us is a thoroughly up to date exposition of the best thought on the subject of medicine in law. Judge Bell has also added several entirely new articles. This edition we commend to our readers as embodying all that is necessary to know on the subject there treated, and every subject is ably treated; and the opening chapter on the importance of a knowledge of medical jurisprudence we commend especially to our younger brethren. The book is offered, too, at a low price. It is handsomely and substantially bound and ought to be in every doctor's library. D.

